# Silencing FOXA1 suppresses inflammation caused by LPS and promotes osteogenic differentiation of periodontal ligament stem cells through the TLR4/MyD88/NF-κB pathway

**DOI:** 10.17305/bb.2024.11367

**Published:** 2024-12-29

**Authors:** Miao He, Yangdong Lin

**Affiliations:** 1 Department of Stomatology, Tianjin First Central Hospital, Nankai District, Tianjin, China

**Keywords:** Forkhead box protein A1, FOXA1, periodontitis, periodontal ligament stem cells, osteogenic differentiation, TLR4/MyD88/NF-κB pathway

## Abstract

Human periodontal ligament stem cells (hPDLSCs) play a critical role in the regeneration of periodontal tissue. Forkhead box protein A1 (FOXA1) has been implicated in the inflammatory mechanisms of various diseases. However, the role of FOXA1 in periodontal inflammation and its effect on the osteogenic differentiation of hPDLSCs remains unclear. In this study, healthy tooth root-derived hPDLSCs were isolated, and flow cytometry was used to detect cell surface markers. Western blot and immunofluorescence analyses were performed to assess FOXA1 levels in different tissues. The levels of inflammatory factors were measured using Western blot and ELISA kits. Alkaline phosphatase (ALP) staining, alizarin red S staining, and Western blot were employed to evaluate the impact of FOXA1 silencing on the osteogenic differentiation of hPDLSCs. Finally, the protein levels in the Toll-like receptor 4 (TLR4)/Myeloid differentiation factor-88 (MyD88)/NF-κB pathway were analyzed using Western blot. Results showed that periodontal membrane tissues from patients with periodontitis exhibited a marked increase in FOXA1 levels. Lipopolysaccharide (LPS) treatment significantly upregulated FOXA1 expression in hPDLSCs, elevated inflammatory factor levels, and inhibited osteogenic differentiation. However, silencing FOXA1 mitigated the effects of LPS. Furthermore, LPS treatment activated the TLR4/MyD88/NF-κB pathway, while FOXA1 silencing impeded this activation. Notably, the application of the TLR4 agonist CRX-527 reversed the inhibitory effects of FOXA1 silencing on LPS-induced responses. In summary, silencing FOXA1 reduced cellular inflammation by inhibiting the TLR4/MyD88/NF-κB pathway and alleviated the suppressive effects of LPS on the osteogenic differentiation of hPDLSCs.

## Introduction

Periodontitis is a chronic inflammatory condition caused by pathogenic microorganisms in dental plaque, affecting the periodontium and the tissues that support teeth. It has a high and steadily increasing global prevalence [1, 2]. Severe periodontitis impacts over 11% of the global population [3]. Clinically, it is characterized by loss of clinical attachment, periodontal pocket formation, red, swollen, and bleeding gums, and alveolar bone loss [4, 5]. If left untreated, the progressive immune-inflammatory response causes irreversible damage to the periodontal support tissues, eventually leading to tooth mobility and loss [6]. The optimal treatment for periodontal tissue loss, as seen in periodontitis, is to achieve some degree of periodontal regeneration [7]. Human periodontal ligament stem cells (hPDLSCs), a type of mesenchymal stem cell (MSC) found in periodontal ligament tissue, exhibit robust proliferative capacity and multidirectional differentiation potential. These cells can regenerate odontoid structures, alveolar bone, and periodontal ligament-like tissues both *in vitro* and *in vivo*, making them key players in periodontal tissue regeneration [8, 9]. However, inflammatory environments negatively impact the osteogenic differentiation potential of hPDLSCs, thereby hindering periodontal tissue regeneration [10]. Studies have shown that reducing lipopolysaccharide (LPS)-induced pro-inflammatory cytokine production and oxidative stress enhances the osteogenic differentiation of hPDLSCs and prevents LPS-induced apoptosis [11]. Therefore, eliminating periodontal inflammation and restoring the regenerative capacity of hPDLSCs is critical.

Forkhead box protein A1 (FOXA1), a member of the Forkhead box family with a conserved DNA-binding domain, plays a vital role in cell growth, differentiation, and embryonic development [12, 13]. FOXA1 is implicated in tumor progression, epithelial–mesenchymal transition, and metabolic processes, significantly influencing cancer cell resistance to chemotherapy [14, 15]. Interestingly, FOXA1 is also involved in inflammatory processes in diseases, such as tumors, neuronal injury, and sepsis-induced kidney injury [16–18]. Notably, Li et al. [19] reported that FOXA1 knockdown promoted osteogenic differentiation in human bone marrow MSCs, increased mineral deposition, and improved femoral defect healing. However, the role of FOXA1 in periodontal inflammation and its effects on the osteogenic differentiation of hPDLSCs remain unclear. The Toll-like receptor 4 (TLR4)/Myeloid differentiation factor-88 (MyD88)/NF-κB pathway is central to inflammation regulation [20, 21]. Tang et al. [22] demonstrated that blocking this pathway reduced LPS-induced periodontal inflammation and promoted tissue repair. Similarly, another study showed that lipoxin A4 exerts anti-inflammatory effects by inhibiting the TLR4/MyD88/NF-κB pathway, thereby reducing LPS-induced inflammation in periodontal ligament cells [23]. In this study, we isolated hPDLSCs from healthy tooth roots to examine FOXA1 expression levels in periodontal tissues and hPDLSCs. We investigated the effects of FOXA1 silencing on inflammation and the osteogenic differentiation of hPDLSCs. Additionally, we explored whether FOXA1 exerts its effects via modulation of the TLR4/MyD88/NF-κB pathway. This research aims to elucidate the precise mechanisms through which FOXA1 impacts hPDLSCs and provide new insights into the treatment of periodontitis.

## Materials and methods

### Clinical tissue samples

Healthy teeth (*n* ═ 6) were collected from orthodontic patients aged 18–45 years who were admitted to Tianjin First Central Hospital for orthodontic extractions. These patients were free of tooth decay, apical periodontitis, periodontitis, and systemic diseases. Additionally, teeth extracted from periodontitis patients (*n* ═ 6) were included. The periodontitis patients had not undergone periodontal scaling within the past three months and were free of systemic diseases. Clinical characteristics of the periodontitis group included a clinical attachment level of 3–4 mm, bone resorption of 15%–30%, and a maximum periodontal probing depth of ≤5 mm. Before extraction, the teeth and their surrounding tissues were thoroughly disinfected. Immediately after extraction, the teeth were placed in DMEM culture solution (12491015, Gibco) containing 200 U/mL penicillin–streptomycin (15140122, Gibco, Grand Island, NY, USA) and stored in an icebox for future use. The Ethics Committee of Tianjin First Central Hospital (approval no. KY-20220902) approved the study, and written informed consent was obtained from all sample donors.

### Isolation and culture of hPDLSCs

One-third of the periodontal membrane tissue from a healthy tooth root was carefully scraped off in a sterile environment using a surgical blade. The tissue block was then cut into 1 mm × 1 mm × 1 mm pieces using ophthalmic scissors. After multiple rinses with PBS buffer, the samples were incubated in a solution containing 3 mg/mL type I collagenase and 4 mg/mL neutral protease for 10 min at 37 ^∘^C. The resulting single-cell suspension was filtered through a 70 µm mesh filter, followed by the addition of DMEM medium containing 15% fetal bovine serum (A5670801, Gibco) and 100 U/mL penicillin–streptomycin. After centrifugation at 800 *g* for 5 min, the supernatant was discarded, and a small amount of complete medium (DMEM with 15% fetal bovine serum and 100 U/mL penicillin–streptomycin) was added. The pellet was gently homogenized using a pipette tip and then inoculated into Petri dishes (101VR20, Thermo Fisher Scientific, Waltham, MA, USA) along with any suspended cells. The following day, an appropriate amount of complete medium was added, and the culture medium was replaced every 2–3 days. When the cells reached approximately 80% confluence, passaging was performed at a 1:3 ratio using enzymatic digestion. For subsequent experiments, the 3rd passage of hPDLSCs was used to avoid potential alterations in cell behavior associated with prolonged passaging [24, 25]. The cell culture was maintained at 37 ^∘^C in a humidified atmosphere with 5% CO_2_.

### Identification of hPDLSCs

The third generation of hPDLSCs was subjected to trypsin digestion and rinsed twice with PBS, after which the cell concentration was adjusted to 1 × 10^ImEquation2^ cells/mL. The following antibodies, obtained from BioLegend (San Diego, CA, USA), were added individually: PE anti-human CD90, PE anti-human CD29, PE anti-human CD146, FITC anti-human CD31, and FITC anti-human CD34. The cells were incubated with the antibodies for 30 min in a dark environment at 4 ^∘^C. After incubation, the cells were rinsed twice with PBS and transferred to flow cytometry-specific tubes. Flow cytometry (BD FACSCalibur™, BD Biosciences, San Jose, CA, USA) was then used to evaluate the surface marker molecules of the hPDLSCs.

### Cell transfection and treatment

The 3rd generation hPDLSCs were cultured to the logarithmic growth phase and seeded into 24-well plates at a density of 1 × 10^ImEquation3^ cells/well. FOXA1 short hairpin RNA (sh-FOXA1) and the negative control (sh-NC) were synthesized by RiboBio Co., Ltd. (Guangzhou, Guangdong, China). Lipofectamine™ 3000 reagent (L3000001, Invitrogen, Carlsbad, CA, USA) was diluted with Opti-MEM™ medium (31985070, Invitrogen). The shRNA premix was prepared by diluting the shRNA with Opti-MEM™ medium, followed by the addition of P3000™ reagent and thorough mixing. Diluted DNA was then combined with the diluted Lipofectamine™ 3000 reagent at a 1:1 ratio. Once the cells reached 70% confluence, the shRNA-lipid complexes were transfected into the cells. After 48 h of incubation, RNA was extracted using Trizol reagent (15596026, Invitrogen). Transfection efficiency was assessed by measuring FOXA1 levels in the cells. To establish a periodontitis cell model, hPDLSCs were treated with LPS at concentrations of 1, 5, or 10 µg/mL (SMB00610, Sigma-Aldrich, St. Louis, MO, USA) for 24 h [26]. For subsequent experiments: LPS group: hPDLSCs were incubated with LPS (10 µg/mL) for 24 h. LPS+sh-NC group: hPDLSCs transfected with sh-NC were treated with LPS (10 µg/mL) for 24 h. LPS+sh-FOXA1 group: hPDLSCs transfected with sh-FOXA1 were treated with LPS (10 µg/mL) for 24 h. LPS+sh-FOXA1+CRX-527 group: hPDLSCs transfected with sh-FOXA1 were pre-treated with the TLR4 agonist CRX-527 (0.5 ng/mL, tlrl-crx527, InvivoGen, San Diego, CA, USA) for 2 h, followed by treatment with LPS (10 µg/mL) for 24 h.

### Alizarin red S staining

The third-generation hPDLSCs were seeded into 6-well culture plates at a density of 2 × 10^ImEquation4^ cells per well and incubated in DMEM medium for two days. Subsequently, the DMEM medium was replaced with osteogenic differentiation medium (HUXDP-90021, Cyagen Biosciences, Suzhou, Jiangsu, China). After 14 days of culture, the hPDLSCs were fixed with 4% paraformaldehyde (P1110, Solarbio, Beijing, China) for 20 min, stained with 2% alizarin red S solution (C0148S, Beyotime, Shanghai, China) for 30 min, and rinsed twice with deionized water. Images of the calcium deposition were captured using a microscope (XK-DZ004, SINICO Optical Instrument Co., Ltd., Shenzhen, China). To quantify the calcium deposition, the calcium nodules were dissolved using 10% cetylpyridinium chloride (52340, Sigma-Aldrich), and the OD562 value was measured with a microplate reader (1410101, Thermo Fisher Scientific).

### Oil red O staining

The third-generation hPDLSCs were seeded into six-well culture plates at a density of 2 × 10^ImEquation5^ cells per well and cultured in DMEM medium for two days. Once cell confluence reached approximately 80%, the DMEM medium was replaced with lipid-induction differentiation medium (HUXXC-90031, Cyagen Biosciences). The medium was refreshed every two days. After 14 days of induction, the process was terminated upon observing lipid droplet formation under a microscope. The cells were then rinsed three times with PBS and fixed with 4% paraformaldehyde for 30 min at room temperature. The Oil Red O staining solution was prepared by mixing Oil Red O stock solution with its diluent in a 3:2 ratio (C0157S, Beyotime). A total of 1 mL of the staining working solution was added to each well to evenly cover the cells, and the cells were stained for 15 min. Lipid droplet formation was observed under an inverted microscope, and images were captured for documentation.

### Western blot

RIPA lysate (P0013B, Beyotime) was used to lyse cells or tissues for protein extraction, and protein concentrations were determined using the BCA kit (P0012, Beyotime). Following gel electrophoresis, the samples were transferred to PVDF membranes (88518, Invitrogen) and blocked for 1.5 h. After rinsing, the membranes were incubated overnight at 4 ^∘^C with one of the following primary antibodies: FOXA1 (PA5-27157, 1:1000, Invitrogen), inducible nitric oxide synthase (iNOS) primary antibody (1:1000, PA1-036, Invitrogen), cyclooxygenase-2 (COX-2) primary antibody (1:100, 35-8200, Invitrogen), runt-related transcription factor 2 (RUNX2) primary antibody (PA5-82787, 1:1000, Invitrogen), osteopontin (OPN) primary antibody (MA5-17180, 1:500, Invitrogen), osteocalcin (OCN) primary antibody (33-5400, 1:100, Invitrogen), TLR4 primary antibody (ab217274, 1:1000, Abcam Inc., Cambridge, UK), MyD88 primary antibody (ab2064, 1:1000, Abcam Inc.), p65 primary antibody (51–0500, 1:100, Invitrogen) or p-p65 primary antibody (ab16502, 1:200, Abcam Inc.). The next day, after three rinses, the membranes were incubated with goat anti-rabbit IgG secondary antibody (31460, 1:10,000, Invitrogen). Chemiluminescent agent ECL (HY-K1005, MedChemExpress, Monmouth Junction, NJ, USA) was applied evenly to the membranes, which were then scanned using a gel imaging system (iBright CL1500, Invitrogen). Grayscale values of the protein bands were analyzed using Image J software (version 1.54h, Wavne Resband, National Institute of Mental Health, USA), with β-actin (MA1-140, 1:5000, Invitrogen) serving as the internal reference.

### Immunofluorescence

The periodontal tissues were exposed to 4% paraformaldehyde for 48 h, then routinely dehydrated, embedded in paraffin, sectioned, deparaffinized in xylene (534056, Sigma-Aldrich), and rehydrated using a gradient ethanolseries. Antigen retrieval was subsequently performed. hPDLSCs were seeded into 12-well plates with slides placed in the wells. After reaching 50%–60% confluence, the cells were rinsed twice with PBS and fixed with 4% paraformaldehyde for 15 min. Both tissue and cell samples were permeabilized with 0.3% Triton X-100 (X100, Sigma-Aldrich) for 10 min, followed by blocking with 5% bovine serum albumin (BSA, V900933, Sigma-Aldrich) for 2 h. The samples were incubated overnight at 4 ^∘^C with either a FOXA1 primary antibody (1:100) or a TLR4 primary antibody (1:200). The next day, a goat anti-rabbit IgG secondary antibody (1:10,000) was added and incubated in darkness at 37 ^∘^C for 1 h. DAPI staining solution (C0065, Solarbio) was then applied, and the samples were incubated in darkness for 10 min. Fluorescence microscopy was used to observe the samples within 1 h after staining. Fluorescence intensity was quantified by processing the images using ImageJ software.

### qRT-PCR

Trizol reagent (15596026, Invitrogen) was used to extract RNA from hPDLSCs. Complementary DNA (cDNA) was synthesized through reverse transcription using AMV reverse transcriptase (2621, TAKARA, Tokyo, Japan). Subsequently, PCR amplification was performed with TB Green^®^ Premix Ex Taq™ II (CN830S, TAKARA). β-actin was used as the internal reference for the analysis, and the relative expression of the target gene was calculated using the 2^−ΔΔCt^ method. The primer sequences used in this experiment are as follows:

FOXA1: F: 5′-GGTTCTGCCGGTAATAGGGG-3′; R: 5′-TCTCCACTCCAGGCCTACTC-3′. 

β-actin: F: 5′-TCCTATGGGAGAACGGCAGA-3′, R: 5′-TCCTTTGTCCCCTGAGCTTG-3′.

### ELISA

The interleukin-6 (IL-6) ELISA Kit (PI330, Beyotime), tumor necrosis factor-α (TNF-α) ELISA Kit (PT518, Beyotime), and interleukin-1β (IL-1β) ELISA Kit (PI305, Beyotime) were used to quantify IL-1β, IL-6, and TNF-α levels in hPDLSCs. After 48 h of routine culture, the hPDLSCs were centrifuged at 500 *g* for 5 min to collect the supernatant. The supernatant was added to an ELISA well plate and incubated for 2 h. Next, the corresponding antibody was added, and the plate was incubated for 1 h. After three washes with washing buffer, the residual washing solution was removed by shaking, and the plate was incubated with horseradish peroxidase-labeled streptavidin for 30 min. The TMB color development solution was then added and incubated for 10 min. Finally, 50 µL of termination solution was added, mixed thoroughly, and the OD450 value was measured to calculate the concentration.

### Alkaline phosphatase (ALP) staining

Following a 2-day culture in DMEM medium, the hPDLSCs were transferred to an osteogenic differentiation medium. After 14 days, the samples were fixed in 4% paraformaldehyde for 30 min and subsequently stained with BCIP/NBT ALP reagent (C3206, Beyotime) for 20 min in the dark. After two rinses with PBS, the samples were photographed and observed under an inverted microscope. Optical density values were then analyzed using ImageJ software.

### Ethical statement

This study was approved by Tianjin FirstCentral Hospital Ethics Committee (No. KY-20220902).

### Statistical analysis

Each experiment was repeated at least three times, and the results were documented as the mean value ± the corresponding standard deviation. SPSS 26.0 software (IBM SPSS Statistics 26) was used to process and analyze the data statistically. Student’s *t*-test was employed to evaluate distinctions between two groups, while ANOVA was applied for comparisons among multiple sub-groups. Prism software (GraphPad 9.0) was used for plotting. A *P < 0.05 was considered statistically significant.

## Results

### Identification of hPDLSCs

**Figure 1.**
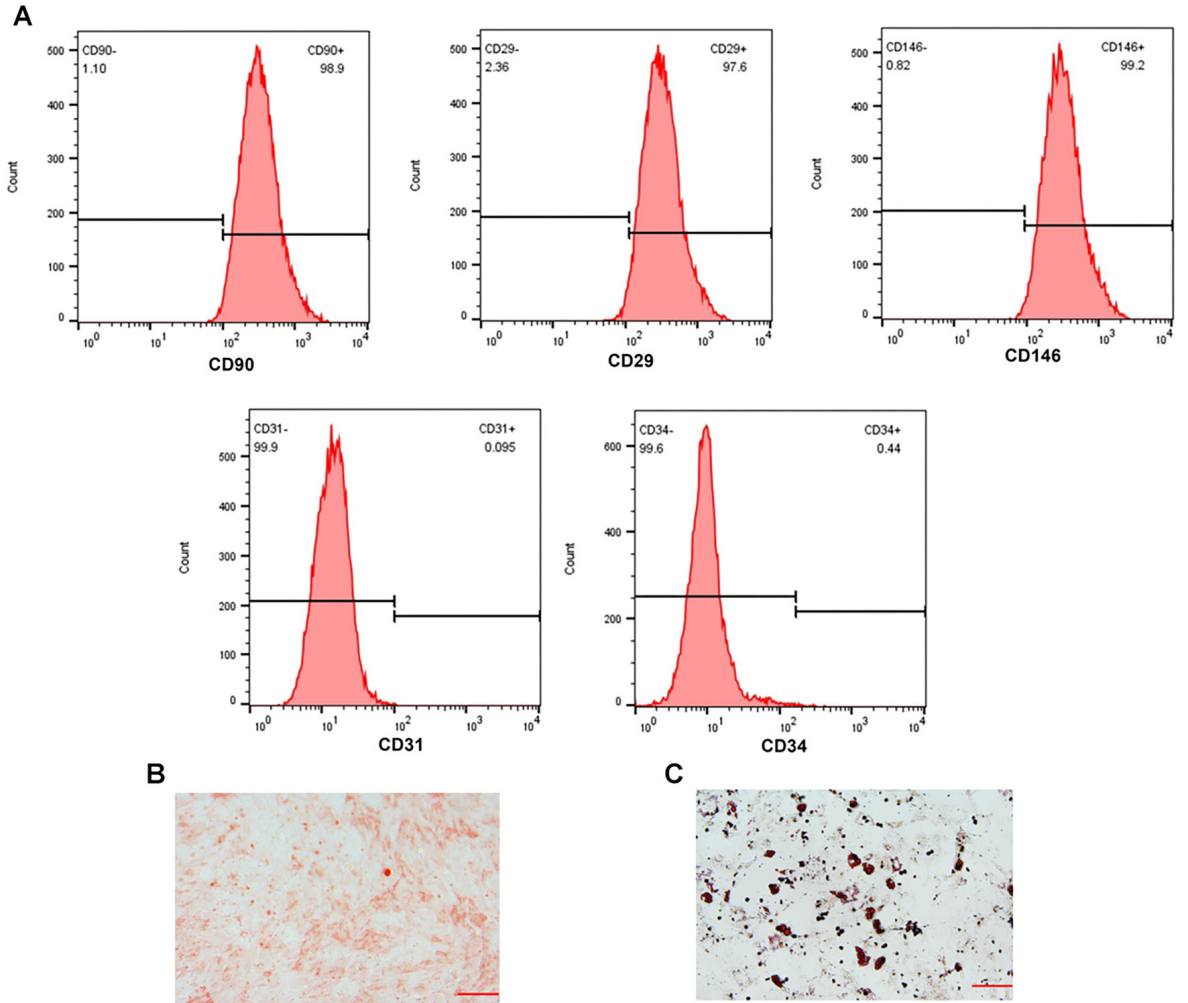
**Identification of hPDLSCs.** (A) The hPDLSCs-associated surface markers were assessed by flow cytometry; (B) Mineralized nodules were identified utilizing alizarin red S staining (20×, bar ═ 100 µm); (C) Lipid droplet formation was determined utilizing Oil red O staining (20×, bar ═ 100 µm). *n* ═ 3. hPDLSC: Human periodontal ligament stem cell.

Flow cytometry was used to characterize hPDLSCs, revealing that these cells positively expressed CD90 (98.90%), CD29 (97.60%), and CD146 (99.20%), while showing negative expression for CD31 (0.10%) and CD34 (0.44%). Notably, CD31 and CD45 are cell surface markers for hematopoietic stem cells, whereas CD90, CD29, and CD146 are markers for MSCs. This indicates that hPDLSCs express MSC markers but lack hematopoietic stem cell markers [27, 28]. Consequently, the cells isolated in this experiment were confirmed to be hPDLSCs (Figure 1A). Additionally, alizarin red S staining demonstrated the presence of deep red mineralized nodules, confirming the osteogenic differentiation potential of hPDLSCs (Figure 1B). Similarly, Oil red O staining revealed orange-red lipid droplets, indicating their capacity for adipogenic differentiation (Figure 1C). These osteogenic and adipogenic induction experiments further verified the multipotent differentiation potential of hPDLSCs.

### FOXA1 expression is upregulated in periodontitis periodontal tissues and LPS-induced hPDLSCs

**Figure 2.**
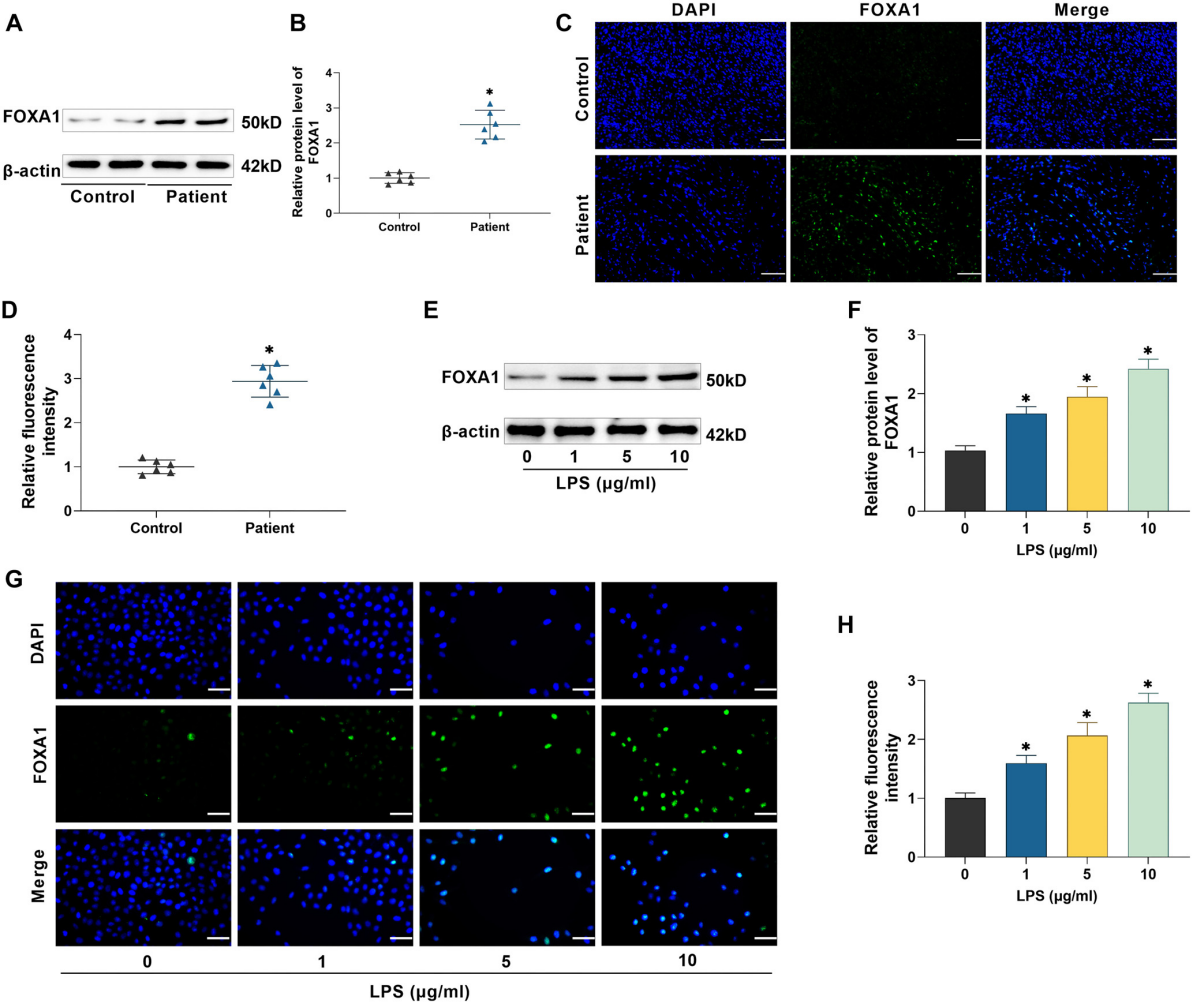
**FOXA1 expression is upregulated in periodontitis periodontal tissues and LPS-induced hPDLSCs.** (A and B) Examining FOXA1 expression in periodontal tissues by Western blot; (C and D) Immunofluorescence was utilized to detect FOXA1 level in periodontal tissues (40×, bar ═ 50 µm) *n* ═ 6; (E and F) Investigating FOXA1 levels in hPDLSCs exposed to varying LPS doses through Western blot analysis; (G and H) Immunofluorescence was utilized to detect FOXA1 levels in hPDLSCs treated with varying LPS doses (40×, bar ═ 50 µm). *n* ═ 3. *P < 0.05. hPDLSC: Human periodontal ligament stem cell; FOXA1: Forkhead box protein A1; LPS: Lipopolysaccharide.

Western blot results revealed a notable rise in the level of FOXA1 protein in the periodontal tissues of periodontitis patients compared to those in healthy tissues (*P* < 0.05) (Figure 2A and 2B). Immunofluorescence results indicated a notable increase in FOXA1 expression in periodontal membrane tissues of periodontitis patients (*P* < 0.05), and this result was consistent with the Western blot results (Figure 2C and 2D). Next, hPDLSCs were exposed to varying doses of LPS (1, 5, and 10 µg/mL) for 24 h, and Western blot findings indicated that the level of FOXA1 protein in hPDLSCs was markedly enhanced in a dose-dependent manner following LPS treatment (*P* < 0.05) (Figure 2E and 2F). Not only that, the immunofluorescence results also indicated a notable rise in FOXA1 expression following LPS treatment (*P* < 0.05) (Figure 2G and 2H). Therefore, in the subsequent experiments, we chose to treat hPDLSCs with LPS at 10 µg/mL.

### Silencing of FOXA1 inhibits LPS-induced inflammation in hPDLSCs

**Figure 3.**
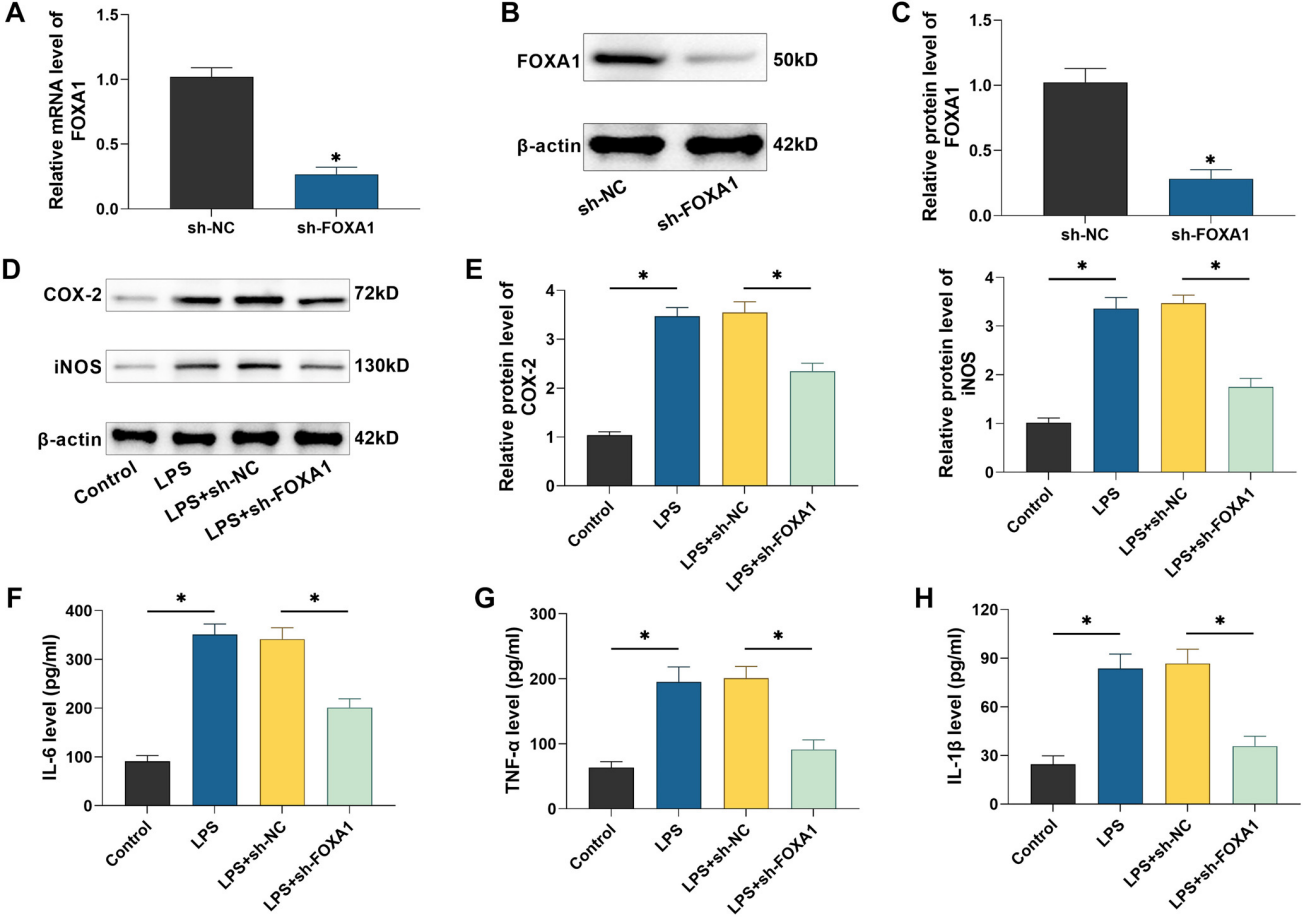
**Silencing FOXA1 suppresses inflammation caused by LPS.** (A–C) Examining FOXA1 levels in hPDLSCs by qRT-PCR and Western blot; (D and E) Examining the levels of COX-2 and iNOS protein in hPDLSCs by Western blot; (F–H) IL-1β, IL-6, and TNF-α contents in hPDLSCs were quantified utilizing ELISA kits. *n* ═ 3. *P < 0.05. hPDLSC: Human periodontal ligament stem cell; FOXA1: Forkhead box protein A1; COX-2: Cyclooxygenase-2; iNOS: Inducible nitric oxide synthase; IL-1β: Interleukin-1β; IL-6: Interleukin-6; TNF- α: Tumor necrosis factor-α; LPS: Lipopolysaccharide.

To investigate the role of FOXA1, we transfected hPDLSCs with sh-FOXA1 and assessed the knockdown efficiency using qRT-PCR and Western blot analysis. The results confirmed a significant reduction in FOXA1 expression in hPDLSCs following transfection with sh-FOXA1, demonstrating its effectiveness for subsequent experiments (*P* < 0.05) (Figure 3A–3C). Next, Western blot analysis was performed to evaluate the levels of inflammatory factors in hPDLSCs. The results showed that LPS treatment significantly increased COX-2 and iNOS protein expression; however, silencing FOXA1 effectively suppressed these LPS-induced increases (*P* < 0.05) (Figure 3D and 3E). Additionally, we used ELISA kits to measure the levels of pro-inflammatory cytokines in hPDLSCs. LPS treatment led to a significant elevation of IL-6, TNF-α, and IL-1β levels (*P* < 0.05). In contrast, FOXA1 silencing markedly reduced these cytokine levels, further indicating that FOXA1 silencing inhibits LPS-induced inflammation in hPDLSCs (Figure 3F–3H).

**Figure 4.**
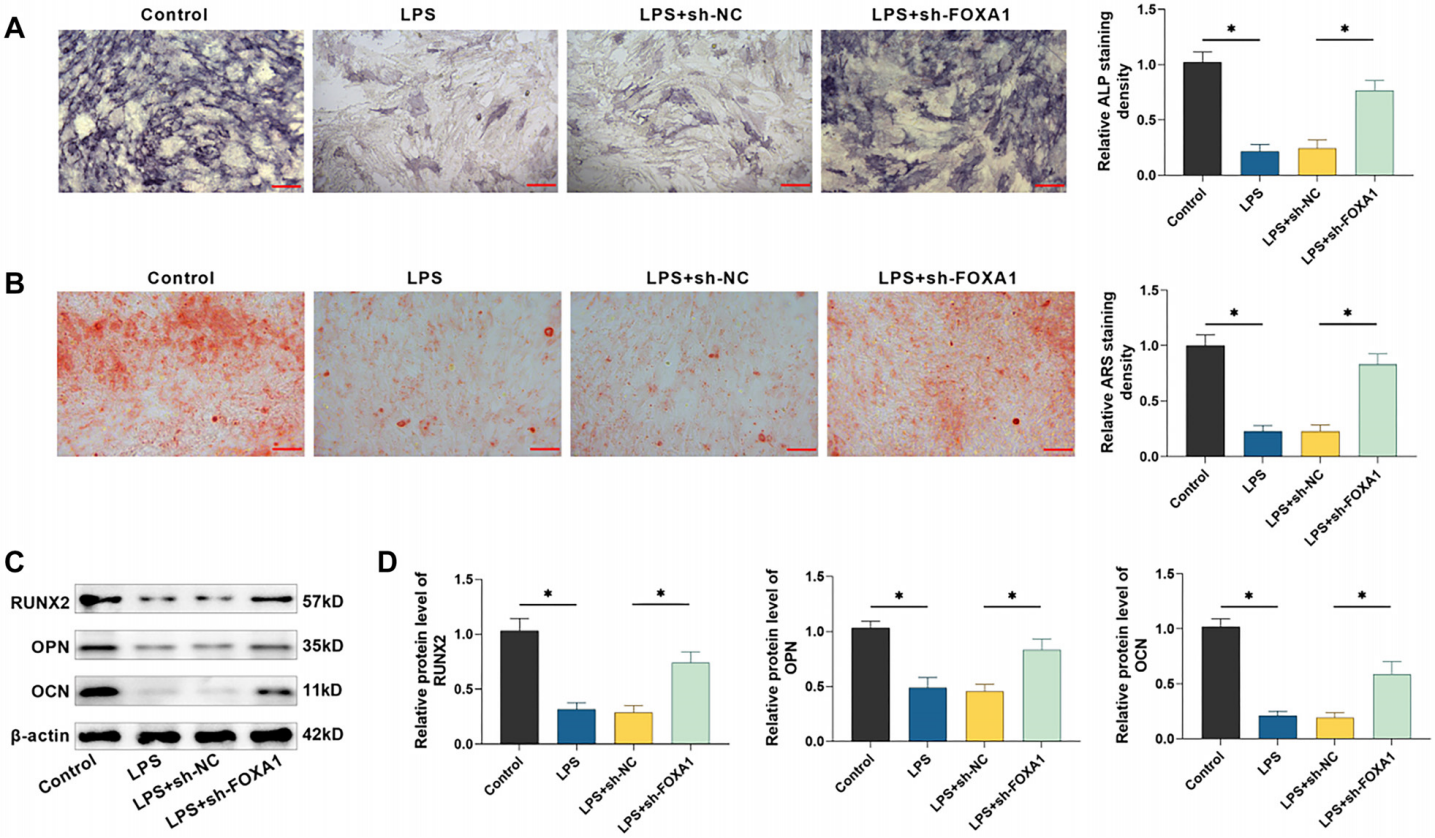
**Silencing FOXA1 attenuates the suppressive influence of LPS on osteogenic differentiation of hPDLSCs.** (A) ALP staining was utilized to detect enzyme activity (20×, bar ═ 100 µm); (B) Mineralized nodules in hPDLSCs were identified utilizing alizarin red S staining (20×, bar ═ 100 µm); (C and D) Examining RUNX2, OPN, and OCN protein levels by Western blot. *n* ═ 3. *P < 0.05. hPDLSC: Human periodontal ligament stem cell; FOXA1: Forkhead box protein A1; LPS: Lipopolysaccharide; RUNX2: Runt-related transcription factor 2; OPN: Osteopontin; OCN: Osteocalcin; ALP: Alkaline phosphatase.

### Silencing of FOXA1 suppresses the suppressive impact of LPS on osteogenic differentiation of hPDLSCs

ALP staining results indicated that LPS treatment resulted in lighter staining of hPDLSCs, fewer cells stained positively for ALP than controls, and silencing of FOXA1 reduced the effect of LPS. Similar to the staining results, ALP quantification results also showed that ALP activity was significantly reduced in hPDLSCs after LPS treatment, and silencing FOXA1 increased ALP activity (*P* < 0.05) (Figure 4A). Additionally, the findings from alizarin red S staining also showed that hPDLSCs in the LPS-treated group produced minimal and scattered mineralized nodules, and the number of mineralized nodules increased significantly after silencing FOXA1. Quantitative assays also showed that LPS inhibited mineralized nodule formation, whereas silencing FOXA1 reduced the inhibition of LPS (*P* < 0.05) (Figure 4B). Not only that, Western blot was utilized to examine the levels of osteogenesis-related proteins in hPDLSCs. The findings revealed a notable reduction in the levels of RUNX2, OPN, and OCN expression in hPDLSCs following LPS treatment (*P* < 0.05), and silencing of FOXA1 attenuated the effect of LPS (Figure 4C and 4D). The results above revealed that LPS treatment reduced the osteogenic differentiation capacity of hPDLSCs, while silencing FOXA1 attenuated the inhibition of LPS.

### Silencing FOXA1 inhibits the TLR4/MyD88/NF-κB pathway

**Figure 5.**
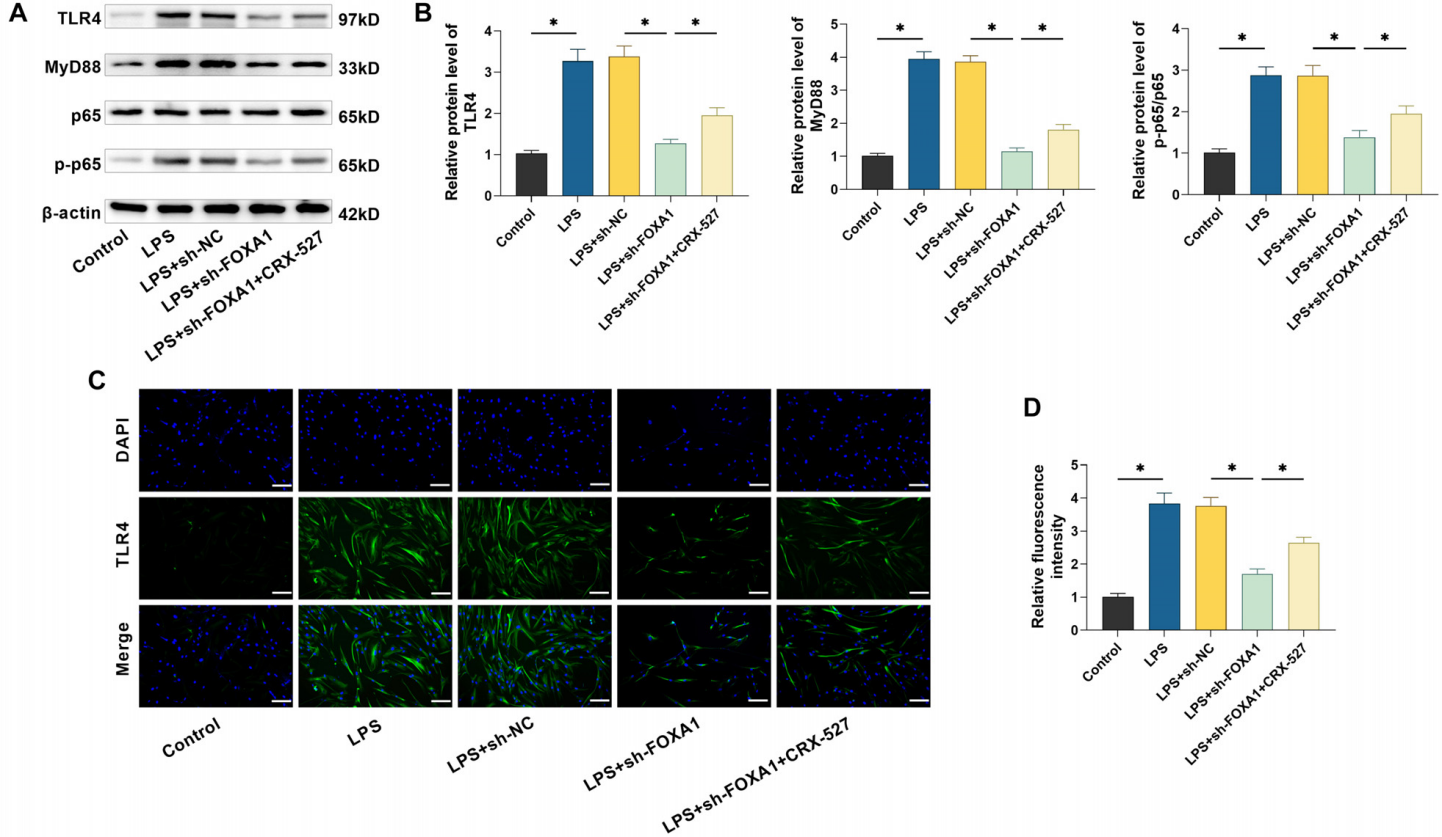
**Silencing FOXA1 hinders TLR4/MyD88/NF-κB pathway in hPDLSCs.** (A and B) Examining the protein levels of TLR4/MyD88/NF-κB pathway through Western blot; (C and D) Immunofluorescence was utilized to determine TLR4 levels in hPDLSCs (20×, bar ═ 100 µm). *n* ═ 3. *P < 0.05. hPDLSC: Human periodontal ligament stem cell; FOXA1: Forkhead box protein A1; LPS: Lipopolysaccharide; TLR4: Toll-like receptor 4; MyD88: Myeloid differentiation factor-88.

To determine the specific mechanisms by which FOXA1 regulates the influences of LPS in hPDLSCs, we examined the levels of TLR4/MyD88/NF-κB pathway-related protein expression. The results revealed a marked rise in the levels of TLR4 and MyD88 protein as well as the phosphorylation of p65 in hPDLSCs following LPS treatment, and silencing of FOXA1 attenuated the effect of LPS. Not only that, we found that intervention with the TLR4 agonist CRX-527 (0.5 ng/mL) was able to attenuate the inhibition of silencing FOXA1 on the effect of LPS action (*P* < 0.05) (Figure 5A and 5B). By immunofluorescence experiments, we observed that TLR4 fluorescence intensity was significantly enhanced in hPDLSCs after LPS treatment, TLR4 fluorescence intensity was notably reduced after silencing of FOXA1 (*P* < 0.05), and the effect of silencing of FOXA1 was attenuated by the CRX-527 intervention (Figure 5C and 5D). The above results indicated that LPS treatment activated the TLR4/MyD88/NF-κB pathway, whereas silencing of FOXA1 inhibited the activation of this pathway.

**Figure 6.**
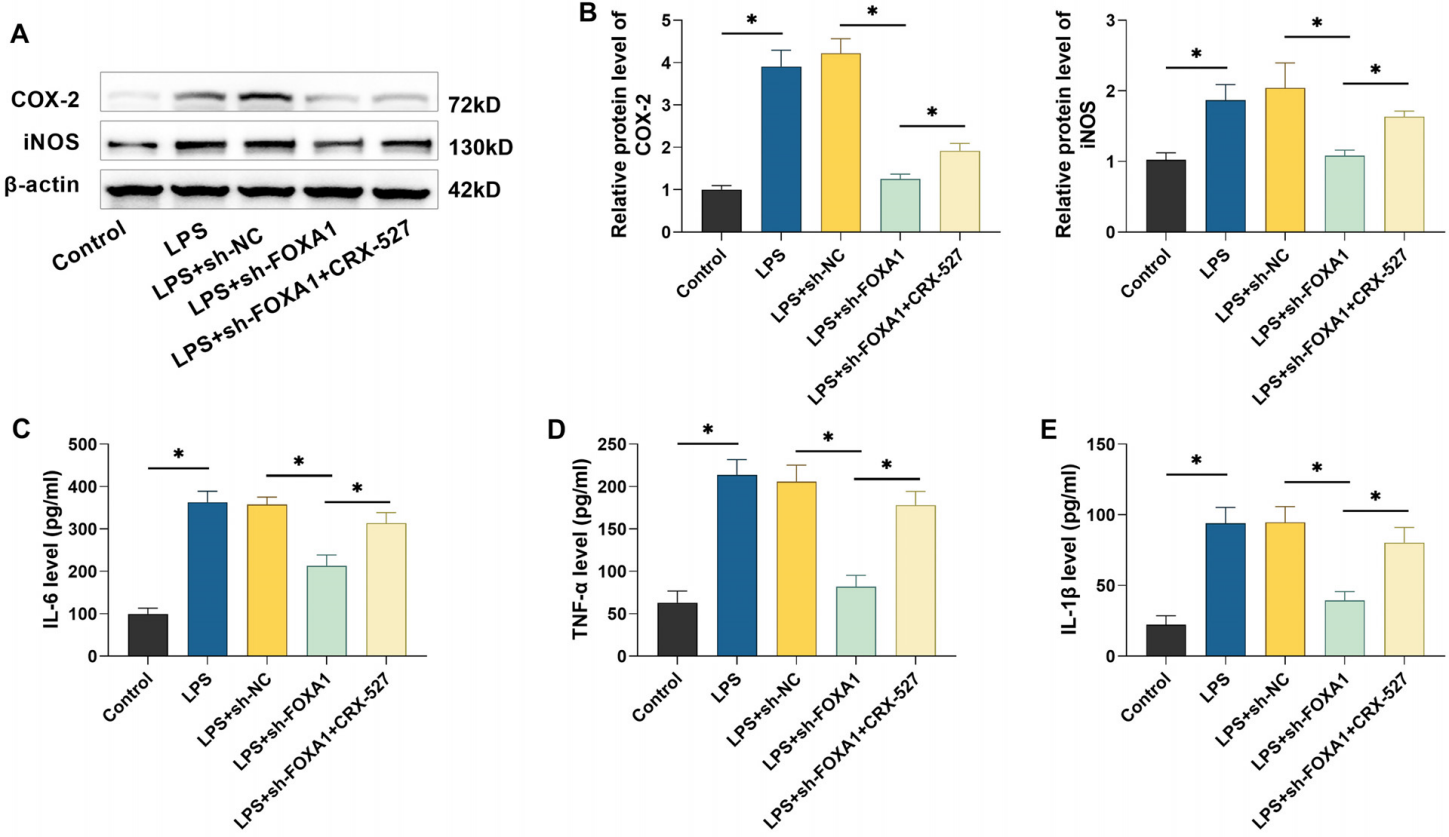
**TLR4 agonist CRX-527 reverses the ameliorative impact ofsilencing FOXA1 on inflammation caused by LPS in hPDLSCs.** (A and B) Examining the levels of COX-2 and iNOS protein in hPDLSCs by Western blot; (C–E) IL-1β, IL-6, and TNF-α contents in hPDLSCs were quantified utilizing ELISA kits. *n* ═ 3. *P < 0.05. hPDLSC: Human periodontal ligament stem cell; FOXA1: Forkhead box protein A1; COX-2: Cyclooxygenase-2; iNOS: Inducible nitric oxide synthase; IL-1β: Interleukin-1β; IL-6: Interleukin-6; TNF-α: Tumor necrosis factor-α; LPS: Lipopolysaccharide; TLR4: Toll-like receptor 4.

### TLR4 agonist CRX-527 reverses the ameliorative effect of silencing FOXA1 on inflammation caused by LPS in hPDLSCs

Next, we investigated whether silencing FOXA1 could inhibit LPS-induced inflammation by targeting the TLR4/MyD88/NF-κB pathway. Silencing FOXA1 significantly reduced the expression of COX-2 and iNOS proteins in hPDLSCs, as demonstrated by Western blot analysis (Figure 6A and 6B). However, the anti-inflammatory effect of FOXA1 silencing was partially reversed by CRX-527 treatment (*P* < 0.05). Additionally, silencing FOXA1 led to a significant decrease in IL-6, TNF-α, and IL-1β levels in hPDLSCs (*P* < 0.05), while CRX-527 intervention mitigated this effect (Figure 6C–6E). These results suggest that silencing FOXA1 may alleviate LPS-induced inflammation in hPDLSCs by disrupting the TLR4/MyD88/NF-κB signaling pathway.

### TLR4 agonist CRX-527 reverses the ameliorative effect of silencing FOXA1 on LPS inhibition of osteogenic differentiation in hPDLSCs

**Figure 7.**
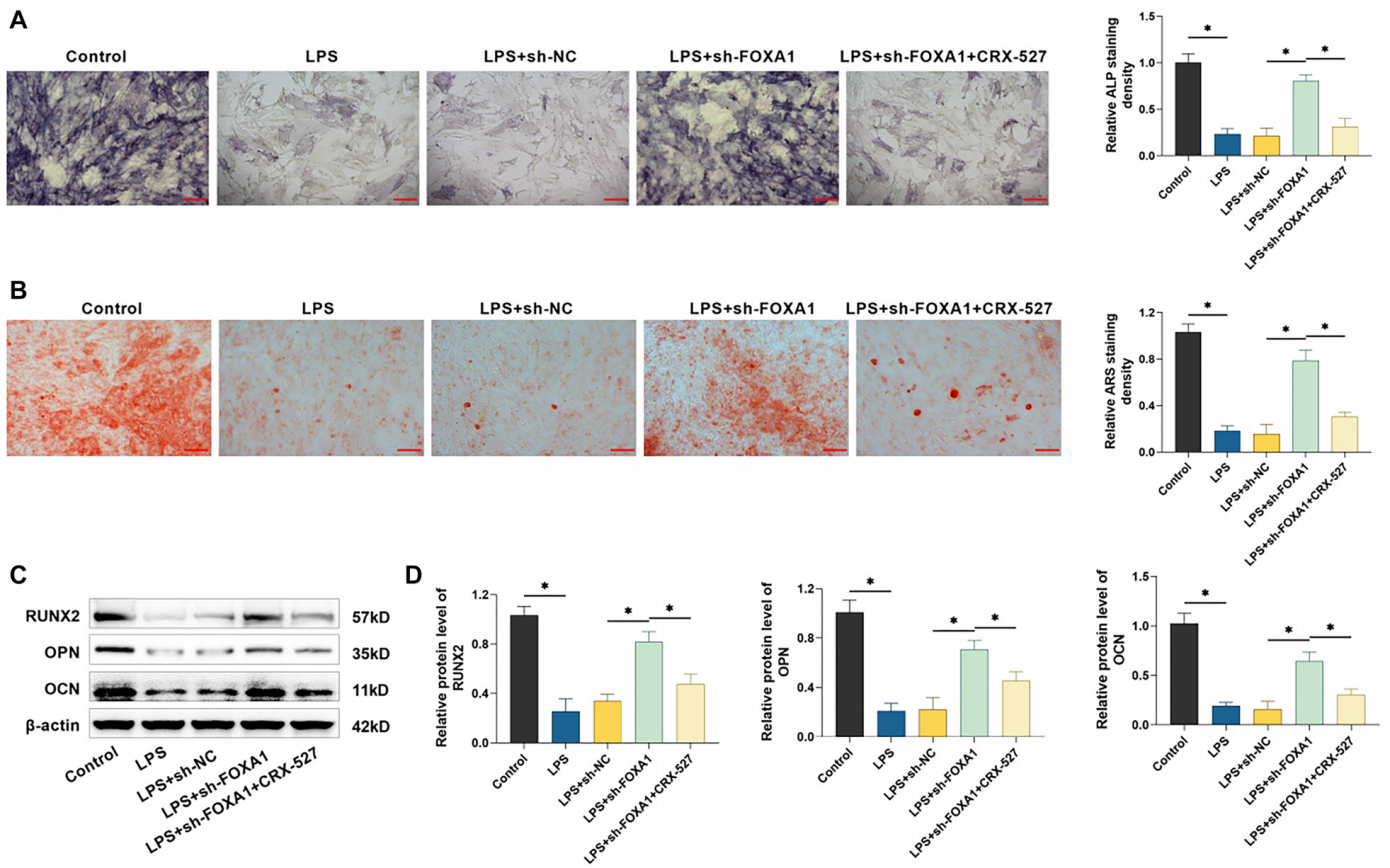
**TLR4 agonist CRX-527 reverses the ameliorative effect of silencing FOXA1 on LPS inhibition of osteogenic differentiation in hPDLSCs.** (A) ALP staining was utilized to detect enzyme activity (20×, bar ═ 100 µm); (B) Mineralized nodules in hPDLSCs were identified utilizing alizarin red S staining (20×, bar ═ 100 µm); (C and D) Examining RUNX2, OPN, and OCN protein levels by Western blot. *n* ═ 3. *P < 0.05. hPDLSC: Human periodontal ligament stem cell; FOXA1: Forkhead box protein A1; LPS: Lipopolysaccharide; TLR4: Toll-like receptor 4; RUNX2: Runt-related transcription factor 2; OPN: Osteopontin; OCN: Osteocalcin; ALP: Alkaline phosphatase.

Finally, we investigated whether silencing FOXA1 enhances osteogenic differentiation by inhibiting the TLR4/MyD88/NF-κB pathway. ALP staining revealed that FOXA1 silencing significantly increased the coloration of hPDLSCs and the number of ALP-staining-positive cells. However, CRX-527 treatment mitigated these effects, reducing ALP activity (*P* < 0.05) (Figure 7A). Similarly, Alizarin Red S staining demonstrated a significant rise in mineralized nodule formation in hPDLSCs after FOXA1 silencing, while CRX-527 reversed this effect, reducing staining intensity (*P* < 0.05) (Figure 7B). Furthermore, Western blot analysis showed that FOXA1 silencing significantly elevated the protein levels of RUNX2, OPN, and OCN in hPDLSCs, while CRX-527 attenuated these increases (*P* < 0.05) (Figure 7C and 7D). These findings suggest that silencing FOXA1 may counteract the suppressive effects of LPS on osteogenic differentiation in hPDLSCs by inhibiting the TLR4/MyD88/NF-κB pathway.

## Discussion

Periodontitis is mainly caused by plaque biofilm, the dynamic equilibrium of periodontal tissues depends on the host’s immune defense and the dynamic equilibrium between microorganisms, microbial dysbiosis breaks the dynamic equilibrium triggering the host’s inflammatory response, which in turn leads to the destruction of periodontal tissues [29, 30]. Several studies have shown that hPDLSCs have the potential to stimulate the regeneration of cementoid, alveolar bone and periodontal membrane-like tissues and have promising clinical applications in the restoration of periodontal defects caused by periodontitis [31, 32]. It has been shown that hPDLSCs express MSC surface markers but not hematopoietic stem cell surface markers, CD31 and CD45 are cell surface markers of hematopoietic stem cells, whereas CD90, CD29, and CD146 are cell surface markers of MSC [27, 28]. In this study, we observed high levels of MSC surface markers CD90, CD29, and CD146 in the isolated cells, while the hematopoietic stem cell surface markers CD31 and CD34 showed low expression, confirming that the cells were indeed hPDLSCs. Not only that, dark red mineralized nodules were visible after alizarin red S staining, and orange-red lipid droplets were visible after Oil red O staining, further confirming the multidirectional differentiation potential of hPDLSCs.

Periodontopathogenic bacteria like Porphyromonas gingivalis can produce substantial amounts of LPS, a key element of gram-negative bacterial endotoxins, which are involved in eliciting an inflammatory response in periodontal tissues [33, 34]. Ni et al. [35] discovered that LPS promotes macrophage M1 polarization and increases the secretion of inflammatory factors. Kukolj et al. [36] reported that LPS can inhibit osteogenesis and enhance lipogenesis and chondrogenesis in hPDLSCs by activating ERK signaling. These researches showed that LPS interfered with the proliferative differentiation and immune functions of hPDLSCs, and could be used to mimic the inflammatory microenvironment in periodontitis [35, 36]. In this study, exposure to LPS caused a rise in inflammatory factors and reduced the levels of osteogenesis-related proteins in hPDLSCs, in line with previous findings. Li and Gou [37] found that FOXA1 was able to exacerbate LPS-induced vascular endothelial cell injury in sepsis by inhibiting NRP2 transcription, whereas silencing of FOXA1 reduced inflammatory factor. In addition, it has been reported that knockdown of FOXA1 enhances osteogenic differentiation of human bone marrow MSCs and improves bone healing [19]. Therefore, we hypothesized that FOXA1 may also play a role in the osteogenic differentiation of hPDLSCs. In patients with periodontitis, FOXA1 expression was found to be notably high in periodontal tissues. Notably, treatment with LPS led to a dose-dependent increase in FOXA1 expression in hPDLSCs, indicating that FOXA1 may be involved in regulating periodontitis progression. We transfected sh-FOXA1 in hPDLSCs and found that silencing FOXA1 attenuated the promotional effect of LPS on inflammation and the inhibitory effect on osteogenic differentiation in hPDLSCs, confirming that FOXA1 plays an important role in periodontitis progression. However, further *in vivo* studies are needed to assess the effect of silencing FOXA1 on periodontitis, which are beyond the scope of the present study.

Recent research findings consistently demonstrate the significant involvement of the TLR pathway in the inflammatory mechanisms of diseases like cancer, myocardial infarction, and atherosclerosis [38–40]. TLR4 is found on a wide range of cells and is responsible for recognizing danger signals and activating immune responses. However, over-activation produces large amounts of pro-inflammatory cytokines and chemokines, disrupting immune homeostasis and accelerating the progression of various inflammatory diseases [41]. Involved in a range of biological functions, NF-κB is a key transcription factor that regulates the innate and adaptive immune responses [42]. TLR4 triggers NF-κB activation via MyD88, promotes the production of inflammatory cytokines and initiates an inflammatory response [43]. It has been reported that LPS-induced upregulation of the TLR4 signaling pathway inhibited osteogenic differentiation and induced adipogenesis in hPDLSCs under inflammatory conditions [25]. Based on the importance of the TLR4/MyD88/NF-κB pathway in inflammation, we investigated whether FOXA1 acts by regulating this pathway. Our findings revealed that LPS treatment activated the TLR4/MyD88/NF-κB pathway, whereas silencing FOXA1 inhibited the activation of this pathway, suggesting that FOXA1 could modulate this pathway. Not only that, the TLR4 agonist CRX-527 intervention was able to attenuate the inhibition of silencing FOXA1 on the effect of LPS, suggesting that silencing FOXA1 acts by hindering the TLR4/MyD88/NF-κB pathway.

## Conclusion

In summary, FOXA1 was significantly overexpressed in periodontal membrane tissues of periodontitis patients, and silencing FOXA1 attenuated LPS-induced cellular inflammation and the inhibition of LPS on osteogenic differentiation of hPDLSCs. Importantly, silencing FOXA1 acts by hindering the TLR4/MyD88/NF-κB pathway. However, this research only elucidated the potential mechanism of action of FOXA1 in affecting hPDLSCs at the cellular level, and further investigation is necessary to explore the impact of FOXA1 in animals. In subsequent studies, we will enhance the design of experimental protocols to investigate the interplay between alveolar bone osteogenesis and osteoclast formation in LPS-induced periodontitis in mice. In conclusion, FOXA1 is a promising therapeutic target for periodontitis and deserves further investigation.

## Supplemental data

### Highlights:

1. FOXA1 is significantly over-expressed in the periodontal tissues of patients suffering from periodontitis.
2. LPS treatment significantly increased FOXA1 expression in hPDLSCs.
3. Silencing of FOXA1 suppressed LPS-induced cellular inflammation.
4. Silencing of FOXA1 reduced the suppressive impact of LPS on the osteogenic differentiation of hPDLSCs.5. Silencing FOXA1 acts by hindering the TLR4/MyD88/NF-κB pathway.

**Graphical abstract**


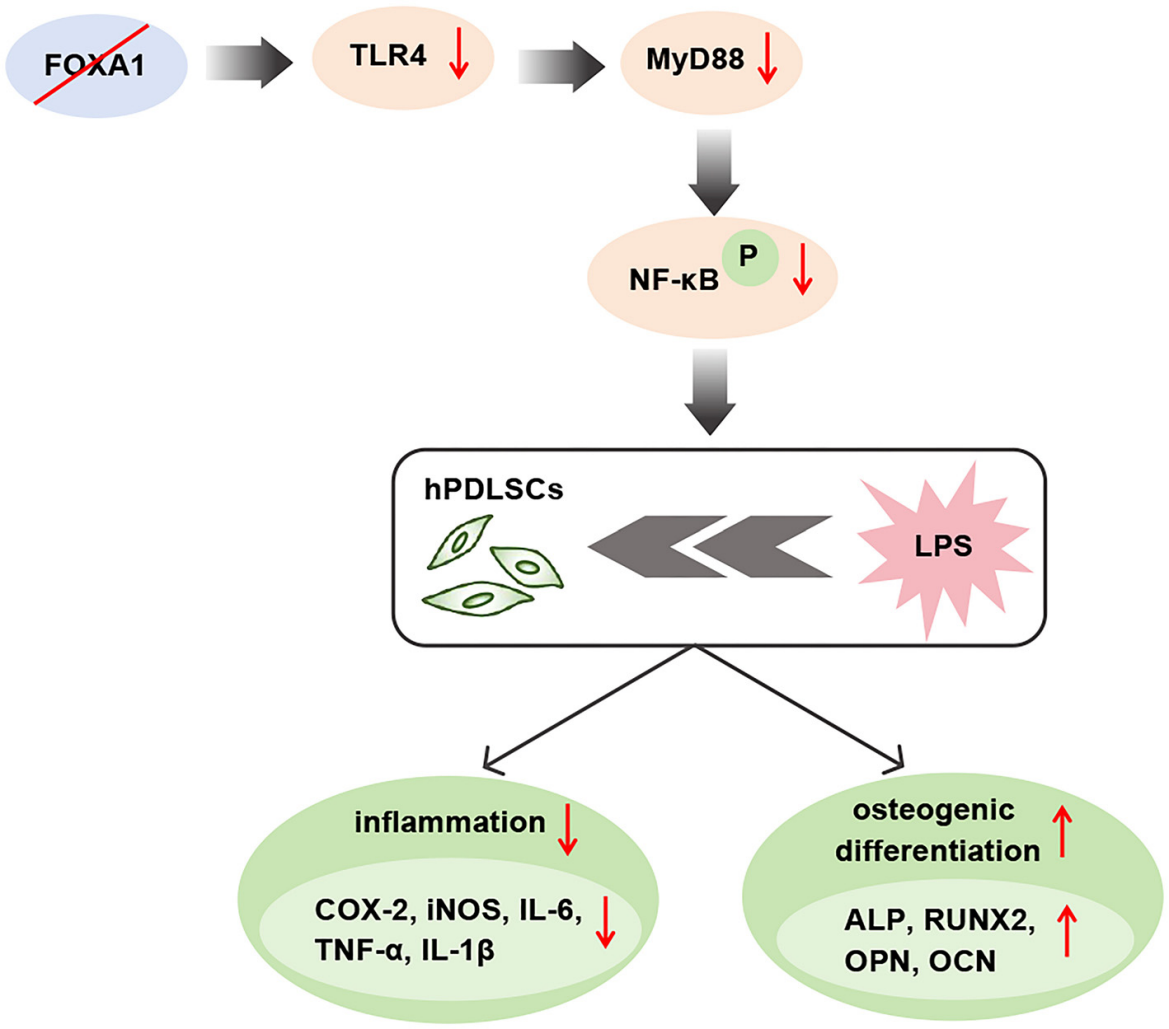


## Data Availability

The corresponding author can provide the data supporting the findings of this study upon request.
